# A Modified Pathological N Stage Including Status of Tumor Deposits in Colorectal Cancer With Nodal Metastasis

**DOI:** 10.3389/fonc.2020.548692

**Published:** 2020-11-11

**Authors:** Jun-Peng Pei, Chun-Dong Zhang, Yu Liang, Cheng Zhang, Kun-Zhe Wu, Yong-Zhi Li, Zhe-Ming Zhao, Dong-Qiu Dai

**Affiliations:** ^1^ Department of Gastrointestinal Surgery, The Fourth Affiliated Hospital of China Medical University, Shenyang, China; ^2^ Department of Gastrointestinal Surgery, Graduate School of Medicine, The University of Tokyo, Tokyo, Japan; ^3^ Cancer Center, The Fourth Affiliated Hospital of China Medical University, Shenyang, China

**Keywords:** colorectal cancer, lymph node, overall survival, prognosis, tumor deposit

## Abstract

**Background:**

The American Joint Committee on Cancer 8th classification states that colorectal cancer (CRC) is classified as N1c stage when regional lymph nodes (LNs) are negative and tumor deposits (TDs) are positive. However, how to classify TDs when regional LNs are positive remains unclear. The current study aimed to investigate the possibility of combining positive LNs and positive TDs to develop a modified pathological N (mpN) stage for CRC.

**Methods:**

We retrospectively analyzed 9,198 patients with stage III CRC from the Surveillance, Epidemiology, and End Results program who underwent surgery (6,440 in the training cohort and 2,758 the validation cohort). The combination of positive LNs and TD status was defined as mpN stage. Overall survival (OS) according to mpN and pathological N (pN) stages was analyzed by the Kaplan–Meier method. The area under the curves (AUCs) and Akaike’s information criterion (AIC) were applied to assess the predictive discrimination abilities and goodness-of-fit of the model. The clinical benefits were measured using decision curve analyses. The validation cohort was used to validate the results.

**Results:**

AUC analysis showed that the prognostic discrimination of mpN stage (AUC = 0.628, 95% confidence interval (CI), 0.616–0.640) was better than that of pN stage (AUC = 0.618, 95% CI, 0.606–0.630, p = 0.006) for OS. The AIC demonstrated that mpN stage (AIC = 30,217) also showed superior model-fitting compared with pN stage (AIC = 30,257) and decision curve analyses revealed that mpN stage had better clinical benefits than pN stage. Similar results were found in the validation cohort.

**Conclusions:**

Among patients with CRC and LN metastasis, mpN stage might be superior to pN stage for assessing prognosis and survival, suggesting that TD status should be included in the pN stage.

## Introduction

Colorectal cancer (CRC) is the third most common malignancy and third-leading cause of cancer-related deaths in the United States (1). The American Joint Committee on Cancer (AJCC) tumor-node-metastasis (TNM) classification has been the most important determinant of prognosis and thus plays a vital role in the management and treatment of patients with CRC. The TNM classifications for CRC have been gradually modified from the 5th to 8th edition, particularly in terms of pathological N (pN) stage, to improve prognosis prediction and guide treatment decision-making (2–5).

Tumor deposits (TDs) are defined as discrete tumor foci in central (or perirectal) fat within the lymphatic drainage cavity of the primary tumor, with no histological evidence of residual lymph node (LN) tissues or vascular structures in the nodules (4). Several recent studies have suggested that TDs are an independent prognostic factor for survival in patients with CRC. Compared with patients without TDs, CRC patients with TDs had a poorer prognosis (6–8). In the absence of regional LN metastasis, the AJCC 7th TNM classification of CRC classified any pathological T stage with positive TDs as N1c stage. This remained unchanged until the current classification (4, 5). However, the latest AJCC 8th TNM classification stipulates that the number of TDs should be recorded, although it remains unclear how TDs should be classified, which could affect the accuracy of CRC staging. This study therefore aimed to investigate how to classify TDs in nodal-positive cases, and to determine the prognostic value of TDs in patients with CRC.

## Materials and Methods

### Patients

This study included patients with CRC who were screened in the Surveillance, Epidemiology, and End Results (SEER) program between 2010 and 2016 (the year of implementation of N1c). The inclusion criteria were: (1) CRC in SEER; (2) essential information available; (3) age 18–75 years; (4) primary and single tumors; (5) nodal-positive cases; (6) underwent surgical treatment; (7) no preoperative therapy; (8) survival > 1 month; and (9) follow-up > 60 months or until death. The exclusion criteria were: (1) no available information; (2) age < 18 or > 75 years; (3) multiple tumors; (4) nodal-negative or stage IV cases; (5) no surgical treatment; (6) received preoperative therapy; (7) postoperative survival < 1 month; and (8) follow-up < 60 months or lost to follow-up.

### Modified pN Stage

pN stage was divided into nine categories based on TD status: pN1aTD–, pN1aTD+, pN1bTD–, pN1bTD+, pN1c, pN2aTD–, pN2aTD+, pN2bTD–, and pN2bTD+. These categories were ranked from lowest to highest hazard ratios (HRs) of overall survival (OS). Log-rank tests were performed to compare consecutive stages, and the four largest of the eight χ^2^ values were identified as the optimal cut-off values. Tumors were then clustered into five substages: mpN1a, mpN1b, mpN2a, mpN2b, and mpN2c.

### Statistical Analyses

Continuous variables were presented as mean ± standard deviation (SD). Kaplan–Meier analysis with log-rank tests was performed. Cox proportional hazards regression model was used to identify independent prognostic factors. The predictive discriminations of the pN and mpN models were assessed by area under the curves (AUCs), and compared using Hanley and McNeil test. Akaike’s information criterion (AIC) was applied to assess the prediction model-fitting (9). Higher AUCs demonstrated superior discrimination and lower AICs indicated better model-fitting. Clinical benefit was further estimated by decision curve analyses (10, 11).

All data were analyzed using SPSS 22.0 statistical package (SPSS Inc., Chicago, IL, USA), MedCalc version 15.2, and R version 3.5.6. All tests were two-sided and a p value < 0.05 was considered statistically significant. A data-use agreement was approved for use of the SEER database. Institutional review board approval was not required because the SEER database holds publicly available de-identified data.

## Results

### Clinicopathologic Features

**Figure 1:**
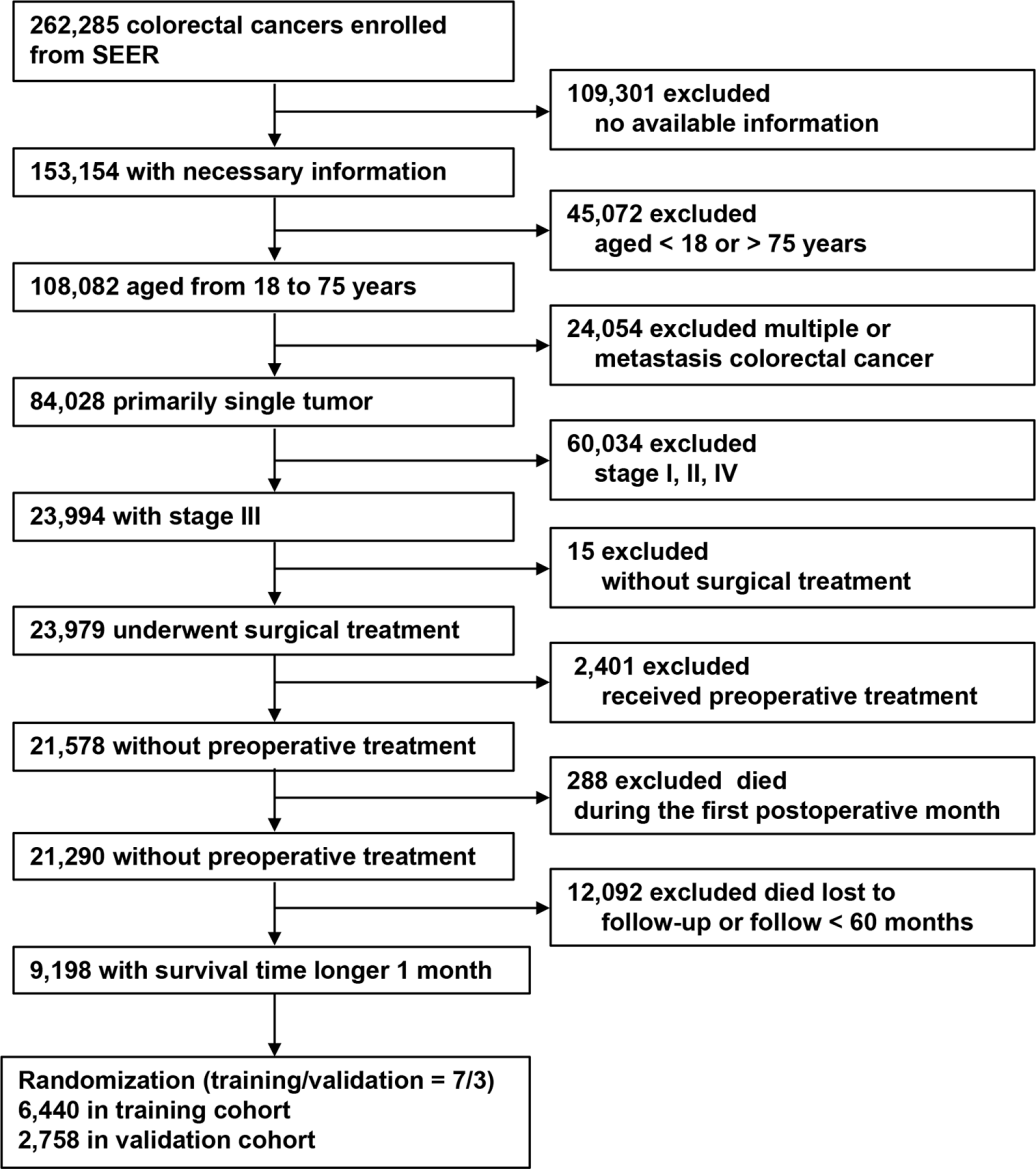
Flow chart for patient selection.

The patient inclusion process is shown in **Figure 1**. A total of 9,198 patients were included and were randomly dividing into a training (70%, n = 6,440) and a validation cohort (30%, n = 2,758). In the training cohort, 5,161 (80.1%) patients had colon cancer and 1,279 (19.9%) had rectal cancer. The mean age (± SD) was 60.0 ± 10.7 years (range, 18–75 years), tumor size was 45.0 ± 2.4 mm (range, 1–200 mm), number of examined LNs was 18.0 ± 10.7 (range, 1–89), and number of positive LNs was 2.0 ± 4.0 (range, 0–89). Positive TDs were observed in 860 cases (13.4%) with a mean of 2.0 ± 5.9 (range, 1–70) TDs per patient (**Table 1**). The clinicopathological characteristics of the validation cohort were similar to the training cohort (**Table 1**).

**Table 1:** Baseline clinicopathologic characteristics and univariate analysis of training and validation cohorts.

Variables	Training cohort^a^			Validation cohort^a^		
	No. of patients (%)	5-Y OS (%)	*P* value	No. of patients (%)	5-Y OS (%)	*P* value^*^
Location			0.450			0.060
Colon	5,161 (80.1)	72.0		2,194 (79.6)	70.1	
Rectum	1,279 (19.9)	73.8		564 (20.4)	74.2	
Sex			0.002			0.039
Female	3,086 (47.9)	74.6		1,301 (47.2)	72.4	
Male	3,354 (52.1)	70.4		1,457 (52.8)	69.6	
Race			0.979			0.656
White	4,853 (75.4)	73.2		2,069 (75)	72.1	
Black	825 (12.8)	64.6		380 (13.8)	62.2	
Other	725 (11.3)	74.5		295 (10.7)	72.6	
Unknown	37 (0.6)	100		14 (0.5)	92.9	
Age, year			<0.001			<0.001
≤60	3,162 (49.1)	77.1		1,337 (48.5)	77.0	
>60	3,278 (50.9)	67.9		1,421 (51.5)	65.3	
Size, cm			<0.001			0.232
≤ 4.5	3,408 (52.9)	74.7		1,477 (53.6)	72.0	
> 4.5	2,755 (42.8)	69.2		1,165 (42.2)	68.8	
Unknown	277 (4.3)	76.1		116 (4.2)	78.4	
Histological grade			<0.001			<0.001
Grade I	355 (5.5)	79.4		138 (5.0)	62.9	
Grade II	4,537 (70.5)	74.9		1,926 (69.8)	74.0	
Grade III	1,236 (19.2)	63.5		551 (20)	64.5	
Grade IV	199 (3.1)	59.6		102 (3.7)	60.1	
Unknown	113 (1.8)	69.8		41 (1.5)	69.1	
pT stage			<0.001			<0.001
pT1	414 (6.4)	90.5		171 (6.2)	84.7	
pT2	745 (11.6)	86.1		276 (10)	88.1	
pT3	4,179 (64.9)	73.3		1,775 (64.4)	72.6	
pT4a	758 (11.8)	55.3		367 (13.3)	53.5	
pT4b	344 (5.3)	47.6		169 (6.1)	49.1	
pN stage			<0.001			<0.001
pN1a	2,079 (32.3)	81.0		909 (33)	78.3	
pN1b	2,030 (31.5)	75.2		826 (29.9)	72.3	
pN1c	273 (4.2)	75.5		140 (5.1)	70.2	
pN2a	1,191 (18.5)	67.6		504 (18.3)	65.5	
pN2b	867 (13.5)	51.1		379 (13.7)	57.7	
TD status			<0.001			<0.001
Negative	5,580 (86.6)	73.8		2,397 (86.9)	72.6	
Positive	860 (13.4)	63.5		361 (13.1)	59.1	
Retrieved LNs			<0.001			<0.001
<12	774 (12.0)	65.0		326 (11.8)	65.0	
≥12	5,688 (88.0)	73.4		2,432 (88.2)	71.7	

No., number; OS, overall survival; pN, pathological N; TD, tumor deposit; Y, year.

^a^Ratio of training to validation cohorts is 7:3 by randomized number using R software.

^*^P values based on chi-squared test.

### Univariate and Multivariate Survival Analyses

The results of univariate and multivariate survival analyses in the training and validation cohorts are shown in **Tables 1** and **2**. Sex, age, tumor size, histological grade, pT stage, pN stage, TD status, and retrieved LNs had significant prognostic impacts on survival in the training cohort according to univariate analysis. Multivariate analysis identified sex, age, histological grade, pT stage, pN stage, TD status, and retrieved LNs as independent prognostic factors for OS.

**Table 2:** Multivariate survival analyses of patients with colorectal cancer in the training and validation cohorts.

Variables	Training cohort			Validation cohort		
	HR	95% CI	^*^P value	HR	95% CI	*P* value^*^
Sex	1.147	1.147 (1.045–1.259)	0.004	1.154	1.154 (1.005–1.325)	0.042
Age, year	1.508	1.508 (1.372–1.658)	<0.001	1.765	1.765 (1.532–2.035)	<0.001
Size, cm	1.068	1.068 (0.983–1.160)	0.120	–	–	–
Histological grade	1.181	1.181 (1.110–1.258)	<0.001	1.094	1.094 (0.992–1.205)	0.071
pT stage	1.574	1.574 (1.487–1.665)	<0.001	1.58	1.58 (1.453–1.718)	<0.001
TD status	1.170	1.170 (1.035–1.323)	0.012	1.375	1.375 (1.147–1.649)	0.001
Retrieved LNs	0.568	0.568 (0.498–0.647)	<0.001	0.675	0.675 (0.554–0.821)	<0.001
pN stage	1.249	1.249 (1.209–1.290)	<0.001	1.174	1.174 (1.120–1.231)	<0.001

CI, confidence interval; HR, hazard ratios; LNs: lymph nodes, mpN, modified pathological N; OS, overall survival; pN, pathological N.

^*^P value based on multivariate Cox proportional hazard models.

Sex, age, histological grade, pT stage, pN stage, TD status, and retrieved LNs had significant impacts on survival in the validation cohort according to univariate analysis, and sex, age, pT stage, pN stage, TD status, and retrieved LNs were also found to be independent prognostic factors for OS by multivariate analysis.

### Prognosis of TDs

**Figure 2:**
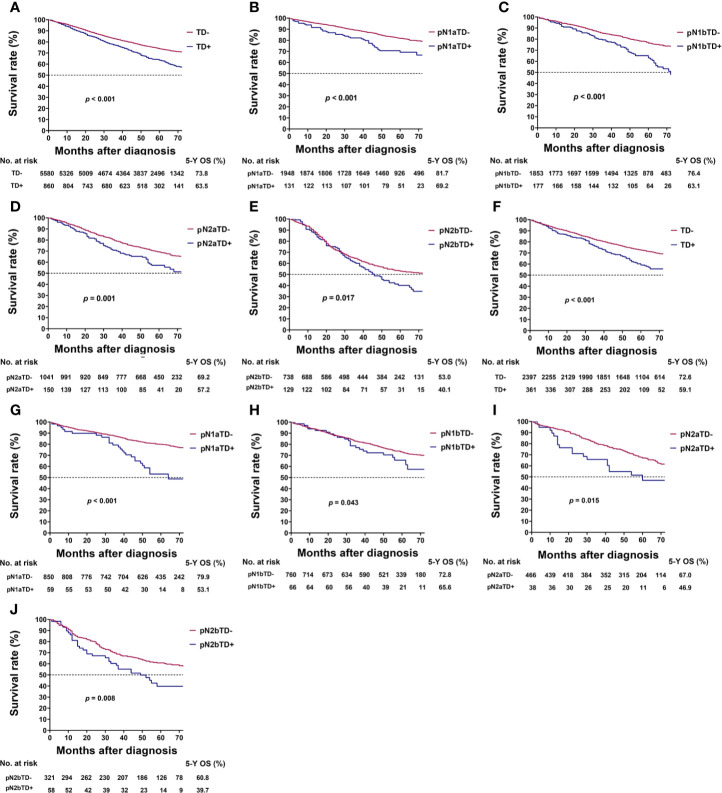
Kaplan–Meier survival curves for patients with and without tumor deposits (TDs) among all patients and pathological N (pN) stage patients. **(A)** All patients in the training cohort; **(B)** pN1a substage in the training cohort; **(C)** pN1b substage in the training cohort; **(D)** pN2a substage in the training cohort; **(E)** pN2b substage in the training cohort; **(F)** all patients in the validation cohort; **(G)** pN1a substage in the validation cohort; **(H)** pN1b substage in the validation cohort; **(I)** pN2a substage in the validation cohort; **(J)** pN2b substage in the validation cohort.

In the training cohort, TD-positive patients had significantly poorer 5-year OS than TD-negative patients (overall patients, 63.5% vs. 73.8%, log-rank test, p < 0.001; **Table 1**, **Figure 2A**). Similar results were found for stages pN1a, pN1b, pN2a, and pN2b (log-rank test, all p < 0.05; **Table 3**, **Figures 2B–E**). These results were confirmed in the validation cohort (log-rank test, all p < 0.05; **Table 3**, **Figures 2F–J**).

**Table 3:** Comparison of 5-year overall survival rates in patients with and without TDs in pN stage.

Variables	TD–		TD+		*P* value^*^
	No. (%)	5-Y OS (%)	No. (%)	5-Y OS (%)	
Training cohort					
pN1a	1,948 (30.2)	81.7	131 (2.0)	69.2	<0.001
pN1b	1,853 (28.8)	76.4	177 (2.7)	63.1	<0.001
pN1c	0	–	273 (4.2)	75.5	–
pN2a	1,041 (16.2)	69.2	150 (2.3)	57.2	0.001
pN2b	738 (11.5)	53.0	129 (2.0)	40.1	0.017
Validation cohort					
pN1a	850 (30.8)	79.9	59 (2.1)	53.1	<0.001
pN1b	760 (27.6)	72.8	66 (2.4)	65.6	0.043
pN1c	0	–	140 (5.1)	70.2	–
pN2a	466 (16.9)	67.0	38 (1.4)	46.9	0.015
pN2b	321 (11.6)	60.8	58 (2.1)	39.7	0.008

No., number; OS, overall survival; pN, pathological N; TD, tumor deposit; Y, year.

^*^P value based on log-rank test, TD+ vs. TD–.

### Modified Pathological N Stage

**Figure 3:**
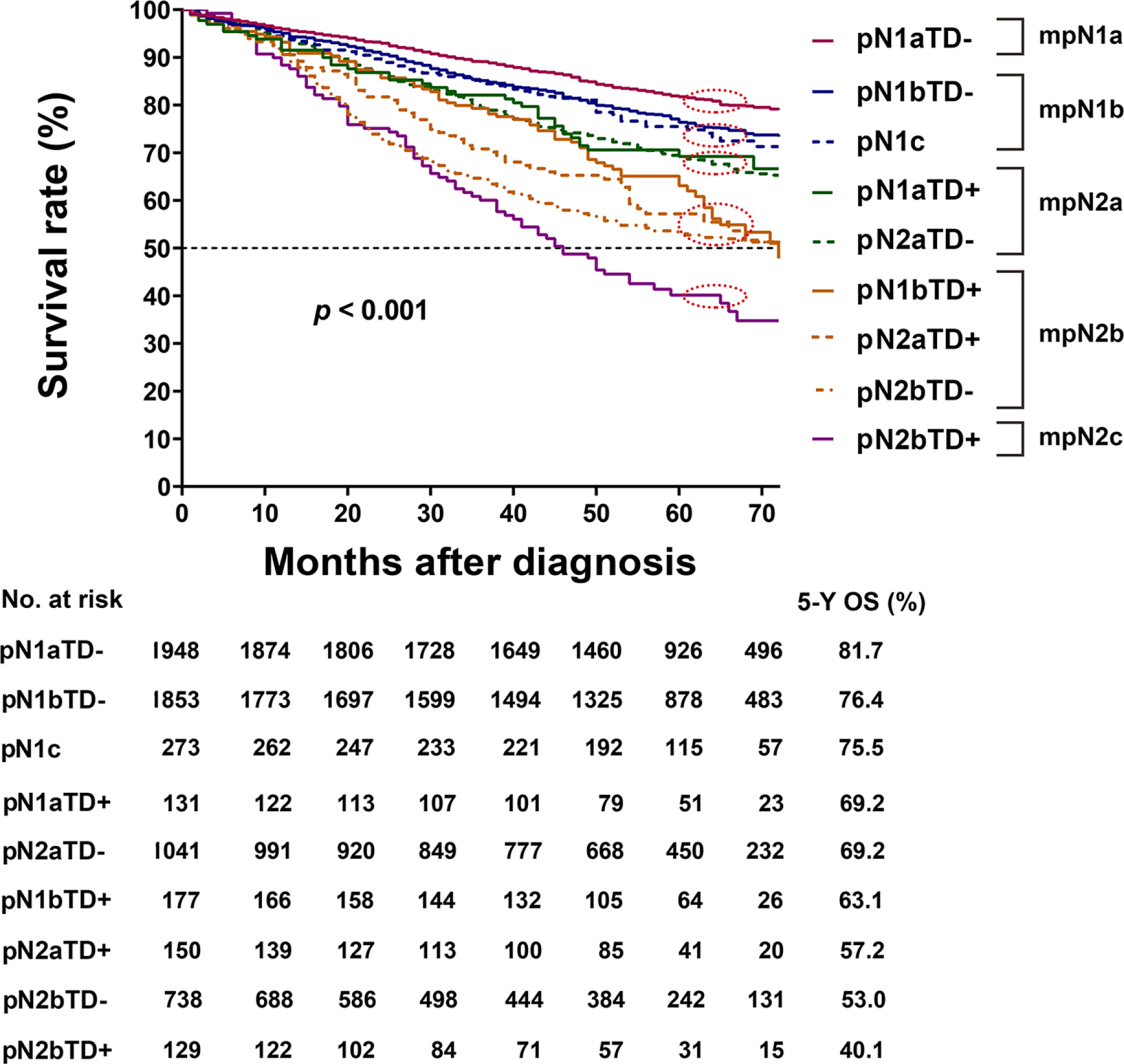
Kaplan–Meier survival curves for pathological N (pN) stage based on the presence or absence of tumor deposits (TDs) in patients in the training cohort.

In the training cohort, there was no significant difference in HRs of overall survival between pN1bTD– and pN1c (HR, 1.317 vs. 1.425, log-rank test, p = 0.522) and between pN1aTD+ and pN2aTD+ (HR, 1.767 vs. 1.822, log-rank test, p = 0.848), and no significant difference between pN2aTD+ and pN1bTD+ (HR, 2.804 vs. 2.421, log-rank test, p = 0.382) or pN2bTD– (HR, 2.804 vs. 3.109, log-rank test, p = 0.402) (**Table 4**, **Figure 3**).

**Table 4:** Modified pN stage in the training cohort.

Variables	HR (95%CI)	3-Y OS (%)	5-Y OS (%)	χ^2^	*P* value^*^	mpN stage
pN1aTD–	1 (Reference)	89.1	81.7	–	–	mpN1a
pN1bTD–	1.317 (1.147–1.513)	85.1	76.4	11.848	<0.001	mpN1b
pN1c	1.425 (1.102–1.844)	84.5	75.5	0.409	0.522	
pN1aTD+	1.767 (1.270–2.458)	82.1	69.2	1.244	0.265	mpN2a
pN2aTD–	1.822 (1.569–2.116)	79.4	69.2	0.037	0.848	
pN1bTD+	2.421 (1.877–3.122)	79.3	63.1	4.896	0.027	mpN2b
pN2aTD+	2.804 (2.153–3.651)	70.8	57.2	0.785	0.382	
pN2bTD–	3.109 (2.682–3.604)	64.4	53.0	0.703	0.402	
pN2bTD+	4.280 (3.346–5.474)	60.0	40.1	5.734	0.017	mpN2c

CI, confidence interval; HR, hazard ratios; mpN, modified pathological N; OS, overall survival; pN, pathological N; Y, year.

^*^P value based on log-rank test.

The categories were ranked from lowest to highest HR of OS as follows: pN1aTD−, pN1bTD−, pN1c, pN1aTD+, pN2aTD–, pN1bTD+, pN2aTD+, pN2bTD−, and pN2bTD+. Based on the four largest χ^2^ values, we modified the pN stage (mpN) as follows: mpN1a (pN1aTD−); mpN1b (pN1bTD− and pN1c); mpN2a (pN1aTD+ and pN2aTD–); mpN2a (pN1bTD+, pN2aTD+, and pN2bTD−); and mpN2c (pN2bTD+) (**Table 4**, **Figure 3**).

### Five-Year OS in Relation to pN and mpN Stages

**Figure 4:**
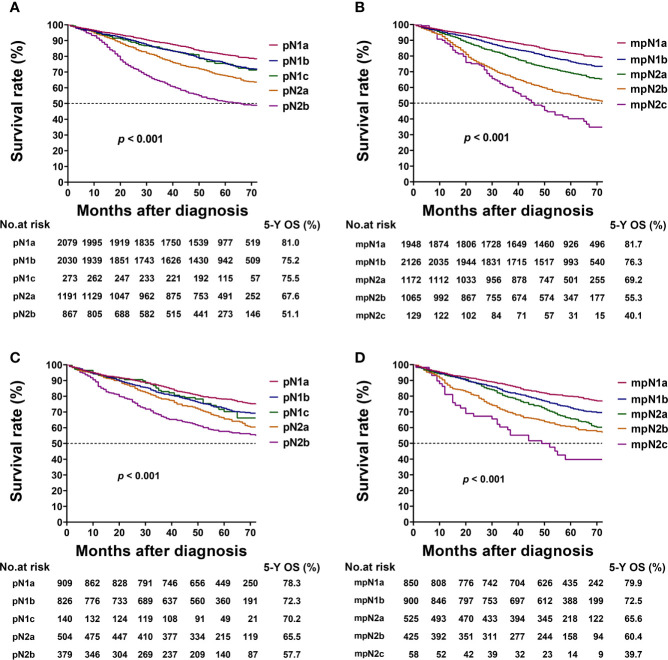
Kaplan–Meier survival curves for 5-year overall survival (OS) based on the pathological N (pN) and modified pN (mpN) stages. Kaplan–Meier survival curves based on **(A)** pN stage in training cohort; **(B)** mpN stage in training cohort; **(C)** pN stage in validation cohort; and **(D)** mpN stage in validation cohort.

In the training cohort, the 5-year OS rates of patients with stages pN1a, pN1b, pN1c, pN2a, and pN2b were 81.0%, 75.2%, 75.5%, 67.6%, and 51.1%, respectively. The 5-year OS of patients with pN1b and pN1c were not significantly different (log-rank test, p = 0.890; **Table 5**, **Figure 4A**).

**Table 5:** Three- and 5-year OS and 95% CI for pN stage and mpN stage in the training and validation cohorts.

Variables	No. of patients (%)	HR (95% CI)	3-Y OS (%)	5-Y OS (%)	*P* value^*^
Training cohort					
pN stage					<0.001
pN1a	2,079 (32.3)	1 (Reference)	88.7	81.0	
pN1b	2,030 (31.5)	1.346 (1.182–1.534)	84.6	75.2	
pN1c	273 (4.2)	1.365 (1.057–1.762)	84.5	75.5	
pN2a	1,191 (18.5)	1.853 (1.612–2.129)	78.3	67.6	
pN2b	867 (13.5)	3.134 (2.733–3.594)	63.7	51.1	
mpN stage					<0.001
mpN1a	1,948 (30.2)	1 (Reference)	89.1	81.7	
mpN1b	2,126 (33)	1.331 (1.163–1.522)	85.0	76.3	
mpN2a	1,172 (18.2)	1.816 (1.570–2.100)	79.7	69.2	
mpN2b	1,065 (16.5)	2.941 (2.566–3.372)	67.8	55.3	
mpN2c	129 (2)	4.279 (3.346–5.474)	60.0	40.1	
Validation cohort					
pN stage					<0.001
pN1a	909 (33)	1 (Reference)	86.4	78.3	
pN1b	826 (29.9)	1.272 (1.055–1.534)	81.9	72.3	
pN1c	140 (5.1)	1.356 (0.966–1.901)	82.9	70.2	
pN2a	504 (18.3)	1.656 (1.357–2.021)	78.3	65.5	
pN2b	379 (13.7)	2.184 (1.779–2.682)	67.2	57.7	
mpN stage					<0.001
mpN1a	850 (30.8)	1 (Reference)	86.9	79.9	
mpN1b	900 (32.6)	1.349 (1.114–1.633)	82.4	72.5	
mpN2a	525 (19)	1.779 (1.451–2.181)	79.3	65.6	
mpN2b	425 (15.4)	2.180 (1.769–2.685)	69.8	60.4	
mpN2c	58 (2.1)	3.674 (2.547–5.300)	58.6	39.7	

CI, confidence interval; HR, hazard ratios; mpN, modified pathological N; No., number; OS, overall survival; pN, pathological N; Y, year.

^*^P value based on log-rank test.

The 5-year OS rates of patients with stages mpN1a, mpN1b, mpN2a, mpN2b, and mpN2c were 81.7%, 76.3%, 69.2%, 55.3%, and 40.1%, respectively. The mpN stage showed enhanced stratification to differentiate between all substages (log-rank test, all p < 0.001; **Table 5**, **Figure 4B**). Similar results were found in the validation cohort (**Table 5**, **Figures 4C, D**).

### Prognostic Value of mpN Stage in Multivariate Analysis

mpN stage and factors significantly associated with OS in univariate analysis were included in multivariate analysis. Sex, age, histological grade, pT stage, retrieved LNs, and mpN stage were identified as independent prognostic factors for OS in the training cohort, and age, pT stage, retrieved LNs, and mpN stage were identified as independent prognostic factors for OS in the validation cohort. TD status and pN stage were not significant prognostic factors in multivariate analysis in either the training cohort (p = 0.064 and 0.872, respectively; **Table 6**) or validation cohort (p = 0.176 and 0.474, respectively; **Table 6**).

**Table 6:** Multivariate survival analysis of patients with colorectal cancer after including mpN stage in the training and validation cohorts.

Prognosis factors	Training cohort			Validation cohort		
	HR	95% CI	^*^P value	HR	95% CI	*P* value^*^
Sex	1.146	1.146 (1.044–1.258)	0.004	1.145	1.145 (0.998–1.315)	0.054
Age, year	1.531	1.531 (1.393–1.683)	<0.001	1.760	1.760 (1.527–2.029)	<0.001
Size, cm	1.076	1.076 (0.990–1.169)	0.085	–	–	–
Histological grade	1.182	1.182 (1.110–1.259)	<0.001	1.091	1.091 (0.990–1.203)	0.078
pT stage	1.572	1.572 (1.485–1.664)	<0.001	1.571	1.571 (1.444 - 1.709)	<0.001
TD status	0.853	0.853 (0.721–1.009)	0.064	1.173	1.173 (0.931–1.477)	0.176
Retrieved LNs	0.553	0.553 (0.485–0.630)	<0.001	0.669	0.669 (0.550–0.815)	<0.001
pN stage	0.994	0.994 (0.920–1.073)	0.872	1.040	1.040 (0.934–1.158)	0.474
mpN stage	1.416	1.416 (1.271–1.578)	<0.001	1.204	1.204 (1.038–1.397)	0.014

CI, confidence interval; HR, hazard ratios; LNs: lymph nodes, mpN, modified pathological N; OS, overall survival; pN, pathological N.

^*^P value based on multivariate Cox proportional hazard models.

### Comparison of Predictive Performances of pN and mpN Stages

**Figure 5:**
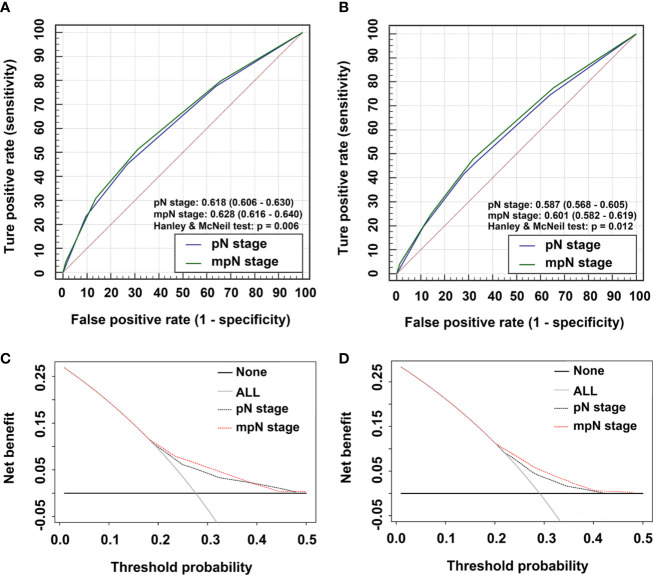
Time-dependent areas under receiver-operating characteristic (ROC) curves (AUC) and Decision curve analysis (DCA) for overall survival (OS). AUCs for **(A)** pathological N (pN) stage and modified pN (mpN) stage in the training cohort and **(B)** pN stage and mpN stage in the validation cohort. DCAs for 5-year OS in **(C)** the training cohort and **(D)** the validation cohort.

In the training cohort, mpN had better prognostic discrimination than pN stage (AUC, 0.628 vs. 0.618, Hanley and McNeil test, p = 0.006) and better model-fitting (AIC, 30,217 vs. 30,257) (**Table 7**, **Figure 5A**). These findings were confirmed in the validation cohort (**Table 7**, **Figure 5B**).

**Table 7:** Prognostic abilities of pN and mpN stages in the training and validation cohorts.

Variables	AUC (95% CI)	AIC	*P* value^*^
Training cohort			0.006
pN stage	0.618 (0.606–0.630)	30,257	
mpN stage	0.628 (0.616–0.640)	30,217	
Validation cohort			0.012
pN stage	0.587 (0.568–0.605)	12,466	
mpN stage	0.601 (0.582–0.619)	12,442	

AUC, Areas under the receiver-operating characteristic curve; AIC, Akaike’s information criterion; CI, confidence interval; mpN, modified pathological N; pN, pathological N.

A higher AUC indicates better discrimination and a lower AIC indicates superior model-fitting;

^*^P value based on Hanley & McNeil test, AUC_pN stage_ vs. AUC_mpN stage_.

### Clinical Use

We evaluated the clinical usefulness of the pN and mpN stages in the training and validation cohorts by decision curve analyses. mpN stage showed higher net benefit than pN stage between threshold probabilities of around 20%–40% in predicting 5-year OS in both the training and validation cohorts (**Figures 5C, D**).

## Discussion

The AJCC TNM classification of CRC is the current standard for tumor staging and thus plays an important role in the management and treatment of patients with CRC. TDs usually occur in subserosal, mesenteric, and nonperitoneal tissues covering the rectum/colon. The concept was first introduced by Gabriel et al. in 1935, and TDs were believed to be the result of the spread of vascular tumors (12). The classification criteria and guidance of how to discriminate TDs from positive LNs have since been revised several times. The AJCC 5th classification defined TDs based on the maximum diameter: nodules < 3 mm were classified as TDs and nodules ≥ 3 mm as positive LNs (2). The AJCC 6th TNM classification defined TDs based on their contours: irregularly contoured nodules were regarded as TDs, while regular smooth nodules were regarded as positive LNs (13). The AJCC 7th TNM classification incorporated TDs into the TNM staging and defined any pT TD-positive but LN-negative lesion as pN1c (4). The pN1c category remained unchanged in the AJCC 8th TNM classification (5); however, TDs are not included in the pN staging for nodal-positive patients, and the validity of the staging in patients with both positive lymph nodes and TDs is controversial. This uncertainty may be because of a lack of sufficient evidence to determine the specific impacts of TDs on the prognosis in patients with CRC.

Previous studies showed that TDs were closely related to a poor prognosis in CRC patients (6, 7, 14–20). The presence of TDs was associated with increased local recurrence and distant metastasis rates (15, 20), and CRC patients with TDs had lower survival rates than those without TDs (6, 7, 14, 16–19), while CRC patients with larger numbers of TDs had poorer prognoses (21). These results suggest that TDs play a significant role in determining the prognosis in patients with CRC.

However, it remains unclear how to classify TDs in the TNM classification. Some researchers have suggested that TDs should be counted as positive LNs, and that this may be superior to the AJCC 7th TNM classification for assessing prognosis in CRC patients (8, 22). However, Frankel et al. indicated that the number of TDs should not be added to the total number of positive LNs (23), while Nagtegaal et al. believed that TDs should not be considered as positive LNs because of their diverse origins (perivascular/intravascular/peritoneal) (24). Furthermore, Basnet et al. demonstrated that TDs were not equivalent to positive LNs, but did not provide detailed information about the exact role of TDs (25). These findings suggest that TDs may differ from positive LNs and may have specific prognostic characteristics. In the current study, we classified patients with stage III CRC according to the presence or absence of TDs and showed that the survival rate of TDs patients was significantly lower than that of patients without TDs, in all pN substages. These results suggested that TDs should be considered as a potential poor prognostic element in CRC patients.

A good classification system should show prognostic discrimination, i.e., the survival rates of each group should be significantly different (26). In the current study, the 5-year OS rates of patients with pN1b and pN1c CRC were not significantly different (75.2% vs 75.5%, log-rank test, p = 0.890); however, mpN stage revealed significant differences in 5-year OS among all five substages (log-rank test, all p < 0.01). mpN stage therefore had a higher discrimination ability than pN stage. To verify the superiority of mpN stage, we included significant prognostic factors in univariate analysis, plus mpN stage, in multivariate survival analysis and showed that mpN stage remained a significant independent prognostic factor; however, TD status and pN stage were not statistically significant. mpN stage was thus a better predictor of prognosis than pN stage. The results of AUC and AIC analyses also showed that mpN stage had higher discriminating and model-fitting abilities than pN stage, and DCA also showed that mpN stage had better clinical benefit than pN stage between threshold probabilities of about 20%–40% in predicting 5-year OS. These results were further confirmed in the validation cohort, suggesting that mpN performed better in terms of predicting prognosis and had higher clinical utility than pN stage in patients with CRC.

However, incorporating TD status into the TNM staging system may have challenges. First, a previous report indicated that approximately 25% of patients with stage III colon cancer had positive TDs (27), while only about 13% of patients with stage III CRC in this study had positive TDs, which was similar to some previous studies based on the SEER database (6, 28). The incidence of TDs is thus variable and depends on the definition and selection criteria used by different researchers (15). This variation is mirrored by the different names used for TDs, including tumor nodules, non-nodal metastatic foci, tumor deposits, extra-nodal foci, extra-bowel skipped cancer infiltration, neoplastic foci, extra-nodal cancer deposits, and mesorectal microfoci. Although the definition of TDs has been revised many times, there is currently no consensus on their exact definition, making it difficult for pathologists to assess the TD status of CRC patients. It is therefore, clinically important to establish a consensus definition of TDs before introducing them into the TNM staging system. In addition, the role of TDs in the spread of CRC has not been fully clarified. Various processes ultimately lead to TDs, and because they are difficult to distinguish, the process responsible for causing them is usually unknown (15). However, lymphatic invasion, lymph node metastases, vascular invasion, and perineural invasion may all lead to TDs, all of which are collectively or individually related to a poor prognosis.

This study had some limitations. First, it showed that the AUC value of mpN stage was significantly higher than that of pN stage, but with a relatively large overlap in 95% CI. Second, this was a large-scale retrospective study, and a lack of rigorous experimental design may have caused selection bias; however, the sample size was large, which might reduce this risk. Third, the findings were based on the population in the United States, and further external validation in other countries is required. Furthermore, although the effect of TDs on disease-free survival of CRC patients is of great importance, we failed to obtain relevant information from the SEER database to allow this to be assessed, and further investigations are therefore required.

## Conclusions

The current study suggests that the presence of TDs is a valuable indicator of a poor prognosis in patients with advanced CRC, particularly in the presence of nodal metastasis. The mpN stage including TD status may provide more accurate prognostic predictions than pN stage, and may thus help clinicians to make better therapeutic decisions. However, the current findings should still be cautious and require further validations by prospective randomized studies.

## Data Availability Statement

The datasets analyzed during this current study are available in SEER database (https://seer.cancer.gov/) to extract the eligible cases. The data are also available from the corresponding author on reasonable request.

## Ethics Statement

Ethics approval and consent was obtained from the Surveillance, Epidemiology, and End Results database.

## Author Contributions

J-PP and C-DZ have contributed equally to this work as co-first authors. J-PP and C-DZ wrote the main text and performed data analysis. J-PP, C-DZ, and D-QD designed the study. J-PP, C-DZ, YL, CZ, K-ZW, Y-ZL, and Z-MZ collected the data. All authors contributed to the article and approved the submitted version.

## Funding

This research was supported in part by the China Scholarship Council, grant number 201908050148.

## Conflict of Interest

The authors declare that the research was conducted in the absence of any commercial or financial relationships that could be construed as a potential conflict of interest.
